# Radiological and pathological findings of a metastatic composite paraganglioma with neuroblastoma in a man: a case report

**DOI:** 10.1186/1752-1947-4-374

**Published:** 2010-11-19

**Authors:** Florian R Fritzsche, Peter K Bode, Sonja Koch, Thomas Frauenfelder

**Affiliations:** 1 Institute of Surgical Pathology, University Hospital Zurich, 8091 Zurich, Switzerland; 2 Clinic of Radiooncology, Kantonsspital Winterthur, 8400 Winterthur, Switzerland; 3 Institute of Diagnostic Radiology, University Hospital Zurich, 8091 Zurich, Switzerland

## Abstract

**Introduction:**

Composite tumors of the adrenal medulla or paraganglia are extremely rare and present a diagnostic dilemma. These tumors consist of a neuroendocrine component mixed with a neural component.

We describe the imaging characteristics together with the corresponding pathological findings of a composite tumor. Apart from any component-specific imaging findings, the hallmark of this entity is the presence of histologically distinguishable components.

**Case presentation:**

A 61-year-old Caucasian man was referred to our hospital due to a suspect lesion found on chest computed tomography carried out for unclear thoracic pain. An abdominal computed tomography scan and ultrasound examination detected a retroperitoneal tumor comprising two different tumor components. Twenty-four-hour urine revealed high levels of normetanephrine, characteristic of a neuroendocrine tumor. An octreoscan prior to surgical procedures revealed multiple osseous and intra-hepatic metastases. The final histopathological workup revealed a composite paraganglioma with neuroblastoma. Our patient died ten months after the initial diagnosis from tumor-associated complications.

**Conclusions:**

Composite paragangliomas with neuroblastoma are rare tumors of the retroperitoneum. Such tumors should be considered in the differential diagnosis of retroperitoneal masses.

## Introduction

Composite tumors of the adrenal medulla or paraganglia are extremely rare. Pheochromocytomas arising from outside the adrenal glands are called paragangliomas. Paragangliomas are more common in the head and neck region than in the retroperitoneum. The synonym mixed neuroendocrine-neural tumor implies that these tumors consist of a neuroendocrine component (paraganglioma or pheochromocytoma) mixed with a neural component (ganglioneuroma, ganglioneuroblastoma, neuroblastoma or peripheral nerve sheath tumor) [1].

We present the ultrasound and computed tomography (CT) findings of a metastatic composite paraganglioma with neuroblastoma presenting as a retroperitoneal mass in correlation with the macroscopic and microscopic pathological findings.

## Case presentation

**Figure 1:**
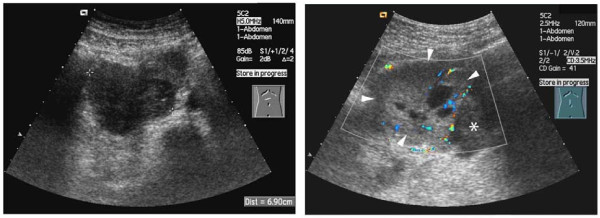
**Ultrasound image of the composite paraganglioma**. (A) The caudally located neuroblastoma. (B) The hypervascularized paraganglioma (arrowhead) and shows the delineation from the neuroblastoma (asterisk).

A 61-year-old Caucasian man underwent a chest CT due to unclear right-sided thoracic pain. In addition our patient complained of abdominal cramps. Examination suggested a retroperitoneal mass seen on the most caudal CT slices. He was referred to our hospital for abdominal ultrasound, showing a 11 cm large retroperitoneal tumor located right and ventral to the abdominal aorta (Figure 1). The craniocaudal dimension extended from the head of pancreas to the aortic bifurcation. The tumor consisted of two different components: the cranial component was well delineated and heterogeneous with hyperechoic and anechoic compartments. The caudal tumor component was poorly delineated, homogeneous and hypo-echoic. The tumor led to a ventral displacement of the duodenum and a compression of the inferior vena cava. Due to an obstruction of the right ureter, there was a right-sided hydronephrosis.

**Figure 2:**
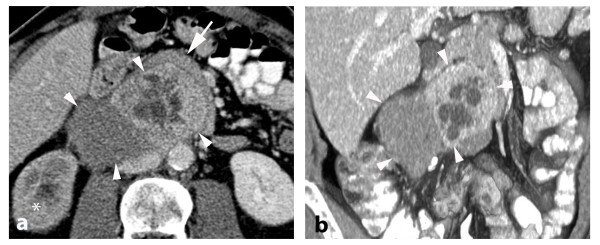
**(A) Axial and (B) coronal multi-planar reformation showing the composite paraganglioma (arrowhead) including the hyperdense paraganglioma with cystic lesions and the hypodense neuroblastoma component compressing the right ureter leading to delayed enhancement of the right kidney (asterisk)**. The duodenum is displaced (arrow).

A subsequent abdominal CT confirmed these findings (Figure 2). As seen by ultrasound, the tumor consisted of two different parts: (1) a well-perfused heterogeneous part with cystic lesions and (2) a less-perfused, homogeneous part. The two parts were well delineated from each other. The tumor partially encased the inferior vena cava, the right common iliac artery and right ureter. In addition the pancreas and the duodenum could not be delineated from the tumor. Due to the obstruction of the right ureter, the right kidney showed delayed enhancement.

Laboratory analyses found elevated levels of normetanephrin (4411 nmol; normal 570 to 1930 nmol) in a 24-hour urine test, clinically proving a neuroendocrine tumor of the pheochromocytoma/paraganglioma family. Unaware of this differential diagnosis, an endosonographic-guided transduodenal fine needle aspiration was performed confirming the diagnosis. Fortunately no hypertensive crisis occurred.

In addition, intra-hepatic metastases were seen on the initial CT scan and a subsequent octreoscan also revealed the presence of intra-osseous metastases. On contrast-enhanced CT the liver metastases had a slight early arterial enhancement with a reduced wash-out during the venous phase. On ultrasound the liver metastases were not well delineated, but appeared slightly hypo-echogenic compared with the surrounding liver tissue. Contrast-enhanced ultrasound was not performed. The CT findings, in particular, would be consistent with metastases from a neuroendocrine tumor.

The presence of metastatic disease precluded a curative resection. However, local resection of the tumor was undertaken for symptomatic relief.

**Figure 3:**
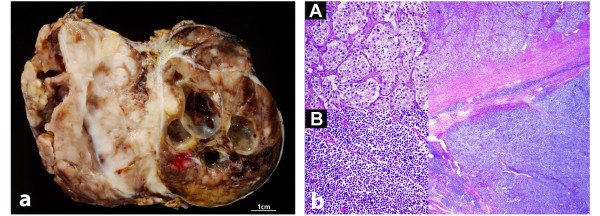
**(A) A macroscopic image of the composite paraganglioma**. The well delineated cranial part (right side) of the tumor with cysts, necrosis and hemorrhages represents the paraganglioma. The less well demarcated, white-gray-tan colored solid part of the tumor (left side) represents the caudally located neuroblastoma component. (B) The microscopic image demonstrates the two histological components of the tumor delineated by fibrous tissue. The paraganglioma (upper part) appears lighter in the low power view corresponding to more abundant cytoplasm of the tumor cells that are arranged in the typical Zellballen pattern (inlet A). The neuroblastoma (lower part) appears bluish corresponding to the densely packed small blue round cells with scant cytoplasm (inlet B). In the fibrous band between both components the vascular invasion of the neuroblastoma can be appreciated.

Macroscopically the partially resected tumor (Figure 3a) reflected the radiological results. The cranial component was well defined and encapsulated and displayed red, brown and black hemorrhagic and cystic areas consistent with the appearance of paragangliomas. Meanwhile the caudal part, corresponding to the neuroblastoma, was macroscopically less well demarcated with a white-gray-tan and solid cut surface.

Microscopically, the encapsulated paraganglioma showed the typical Zellballen growth pattern, an elevated mitotic activity (Ki-67) of up to 50%, necrosis and vascular invasion. The small blue round cells of the neuroblastoma component displayed a highly proliferative (around 90%) and broadly infiltrative growth pattern and lymphovascular invasion was seen (Figure 3b). Immunohistochemically, both components were positive for synaptophysin and somatostatin receptor 2 with the latter one being consistent with the positive octreotid scan. In contrast to the neuroblastoma, the paraganglioma expressed the typical markers chromogranin A and vimentin.

There was no evidence for an amplification of the prognostic oncogene N-myc. Tumor metastasis of the neuroblastoma component was histologically confirmed by lymph node and skin biopsies.

Subsequently, our patient was treated with palliative chemotherapy and radiotherapy beginning with three cycles of carboplatin aqueous solution and etoposide phosphate. On tumor progression palliative radiotherapy with 10 × 3 Gray at multiple locations followed. Subsequently chemotherapy with CHOP (cyclophoshamide, hydroxydaunorubicin, oncovin, prednisone) was started and finally (after two months) changed to a weekly dose of docetaxel with prednisone. Ten months after the initial diagnosis our patient died of cancer-related pulmonary embolism and pneumonia.

## Discussion

The paraganglia are widely dispersed collections of specialized crest cells that lie adjacent to the sympathetic ganglia and plexuses throughout the body [2]. Tumors that arise from chromaffin cells of the adrenal medulla are called pheochromocytomas, whereas those that occur in paraganglia at other sites are called paragangliomas.

Pheochromocytomas or paragangliomas can occur sporadically or in association with inherited conditions (MEN type II, von-Hippel-Lindau syndrome, neurofibromatosis type I). Sporadic forms are usually diagnosed at age 40 to 50, whereas hereditary forms are diagnosed earlier [3,4].

The clinical manifestations of pheochromocytoma result from the known physiologic effects of catecholamine release. The classic triad of headache, palpitation, and excessive sweating is seen during the paroxysmal hypertensive crisis. Urinary normetanephrine or vanillylmandelic acid levels are elevated in over 90% of patients from whom 24-hour urine collections are obtained [5]. Recent data suggest that the false positive rate is lower for vanillylmandelic acid than for metanephrines [6].

If laboratory test results indicate a pheochromocytoma, CT imaging of the adrenal gland as well as of the organ of Zuckerkandl, to encompass all chromaffin cell-bearing tissue along the lower abdominal aorta from the origin of the inferior mesenteric artery to the aortic bifurcation and into the iliac vessels, is often helpful to locate the tumor. On CT, both pheochromocytomas and paragangliomas usually measure 3 cm or larger, demonstrate areas of necrosis or hemorrhage, and may even contain fluid. Due to the danger of a hypertensive crisis, suspected paragangliomas/pheochromocytomas should not be biopsied prior to surgery.

Generally, paragangliomas have a more aggressive course than their adrenal counterparts. Dissemination occurs via both the lymphatic and hematogenous routes, with the most common sites of metastasis being the regional lymph nodes, bone, liver, and lung [7]. With the exception of the presence of distant metastases, it is not possible to differentiate benign from malignant paragangliomas confidently with imaging alone. However, features more frequently noted in malignant tumors are greater tumor weight, confluent necrosis, and the presence of vascular invasion and/or extensive local invasion.

Neuroblastomas are malignant tumors that consist of primitive neuroblasts and may arise anywhere within the sympathetic plexus or adrenal medulla. Two-thirds of neuroblastomas are located in the abdomen, and approximately two-thirds of these abdominal lesions arise in the adrenal gland [7]. Neuroblastomas are more aggressive than ganglioneuromas. Sometimes they invade adjacent organs or encase adjacent vessels. The majority of tumors are irregularly shaped, lobulated, and not encapsulated. On CT, small neuroblastomas may be homogeneous, while larger ones tend to be more heterogeneous owing to tumor necrosis, hemorrhage and calcification [7]. Magnetic resonance imaging (MRI) can be used to help locate a paraganglioma; however, only about 80% of T2-weighted MRI studies will show the characteristic uniform high-signal-intensity image because the presence of internal hemorrhage can reduce signal intensity [7].

In composite paragangliomas, a less-differentiated neuronal component seems to be the leading prognostic feature since metastases occur more often from this component. Accordingly, in our case the metastases were of neuroblastoma-type. Both, the neuroendocrine and the neuronal component are thought to be derived from common chromaffin precursor cells by aberrant differentiation. A deletion of the succinate dehydrogenase subunit B gene has recently been associated with composite paraganglioma with neuroblastoma [8].

On CT, the appearance of the paraganglioma was characterized by relatively sharp outlines and intratumoral heterogeneity with anechogenic lesions, hypoechogenic components, small calcifications and hypervascularization corresponding to the blood-filled cysts and necrotic debris in the macroscopic section. The neuroblastoma was irregularly shaped, lobulated, and not encapsulated.

Having said this, the different possible components of composite paragangliomas clearly imply that these tumors cannot be defined by a single specific imaging pattern but rather by the existence of such different components which subsequently can be correlated to certain morphologic tumor subtypes. As in our case, laboratory data could be of great differential diagnostic help.

Little information is available about the outcome of composite paragangliomas because of their rarity. Some reports have described indolent behavior [1]. However, metastases have been reported in composite paraganglioma with ganglioneuroma [9]. On the other hand, biologic and pathologic predictors of outcome in neuroblastic tumors have been studied extensively during the past decades. It is well recognized that the presence of N-myc amplification is an unfavorable prognostic feature in neuroblastoma. Other unfavorable prognostic indicators for neuroblastic tumors include age and stage at diagnosis, histologic subtype, mitotic-karyorrhexis index, and a variety of other cytogenetic and molecular genetic features [10].

The treatment of these patients includes surgery and chemotherapy according to the most aggressive tumor component as well as prolonged follow-up due to possible late metastases.

## Conclusions

Composite paragangliomas with neuroblastoma are rare tumors of the retroperitoneum. However, such tumors should be considered in the differential diagnosis of retroperitoneal masses. Imaging does not allow a differentiation between benign and malignant tumors, but may assist in pre-operative planning. As these tumors are very rare, there is only limited knowledge about treatment and outcome. In the absence of metastases a resection should be considered.

## Competing interests

The authors declare that they have no competing interests.

## Authors' contributions

TF and SK analyzed the clinical and radiological data. FRF and PKB analyzed and interpreted the pathological data. TF and FRF wrote the main parts of the manuscript. All authors read and approved the final manuscript.

## Consent

Written informed consent was obtained from the patient's next of kin for the publication of this case report and any accompanying images. A copy of the written consent is available for review by the Editor-in-Chief of this journal.
